# The Complex Interaction between the Tumor Micro-Environment and Immune Checkpoints in Breast Cancer

**DOI:** 10.3390/cancers11081205

**Published:** 2019-08-19

**Authors:** Vanessa Barriga, Nyanbol Kuol, Kulmira Nurgali, Vasso Apostolopoulos

**Affiliations:** 1 College of Health and Biomedicine, Victoria University, Melbourne 3030, Australia; 2 Institute for Health and Sport, Victoria University, Melbourne 3030, Australia

**Keywords:** breast cancer, checkpoint, checkpoint inhibitors, tumor microenvironment, tumor-associated macrophages, cancer-associated fibroblasts, tumor-associated neutrophils, IDO, PD-L1, Siglec-9

## Abstract

The progression of breast cancer and its association with clinical outcome and treatment remain largely unexplored. Accumulating data has highlighted the interaction between cells of the immune system and the tumor microenvironment in cancer progression, and although studies have identified multiple facets of cancer progression within the development of the tumor microenvironment (TME) and its constituents, there is lack of research into the associations between breast cancer subtype and staging. Current literature has provided insight into the cells and pathways associated with breast cancer progression through expression analysis. However, there is lack of co-expression studies between immune pathways and cells of the TME that form pro-tumorigenic relationships contributing to immune-evasion. We focus on the immune checkpoint and TME elements that influence cancer progression, particularly studies in molecular subtypes of breast cancer.

## 1. Introduction

Breast carcinoma remains a complex heterogeneous disorder, being the most frequent malignant disease in women worldwide, leading to premature death in women in developed countries [1]. In fact, the higher developed countries such as Europe, Australia, and the USA have the highest incidence of breast cancer compared to those from mid-developed countries including, Latin America, central Europe, and the Caribbean; the lowest incidence being in the low developed countries such as Asia and Africa [2]. In the last decade, the mortality rate of breast cancer has declined as a result of early detection and effective treatments [3,4], although, 20% of women diagnosed with breast cancer will still die from the disease [5]. One of the challenges of breast cancer research has been the characterization of the molecular changes that are associated with breast cancer progression [6]. The diagnosis of breast cancer is based on three key parameters including histological type, tumor grade, and tumor stage. Within this, there are at least 20 different histological subtypes of breast cancer, which display differences in morphology and growth patterns [7].

The main biological markers utilized in the diagnosis and treatment of breast cancer include the overexpression of estrogen receptor (ER), progesterone receptor (PR), and human epidermal growth factor receptor 2 (HER2). Based on these clinically used markers, 5 major molecular intrinsic subtypes of breast cancer have been identified: (i) luminal A (histological phenotype: ER+, PR+, HER2−, Ki67−), (ii) luminal B (ER+. PR+, HER2+/−, Ki67+), (iii) HER2 enriched (ER−, PR−, HER2+), (iv) basal-like subtype (ER−, PR−, HER2−, triple negative; associated with poor prognosis and early onset of metastasis) and (v) claudin-low tumor subtype which is commonly classified as basal-like (or normal breast-like) [8,9,10,11]. In addition to these established subtypes, breast cancer staging refers to the extent of disease and is a critical factor in the prognostic outcomes of the patients as well as for oncologists in making treatment decisions. Staging is categorized by stages 0–IV which is determined by tumor size, invasion of tumor cells into the chest wall and lymph nodes, and distant metastasis.

The proliferation of tumor cells and metastasis is influenced by immune cells present within the tumor microenvironment (TME) and their mechanisms of actions. As such, lymphocytes, fibroblasts, neutrophils, eosinophils, macrophages and myeloid-derived suppressor cells are present within the TME with altered mechanisms and influence the outcome of cancer cell growth and migration. In addition, immune-checkpoints have been identified and can be expressed on both immune cells and tumor cells. A number of negative regulatory mechanisms can inhibit the anti-tumor immune response through the expression of these immune checkpoints. An ongoing immune response can be represented by the upregulation of immune checkpoints (CTLA4, PD-1/PD-L1, IDO and members of the CD33-like siglecs i.e., siglec-7 and siglec-9, but their expression on tumor cells can also be driven by oncogenic pathways. By activating these inhibitory pathways, cancer cells are able to escape from immune cell attack by causing apoptosis of tumor-specific T cells [12,13,14,15,16]. Siglec-7 inhibits NK cell lysis [17] and the mucin MUC1 modulates the tumor immunological microenvironment through engagement of the lectin Siglec-9 [18]. Development of immune checkpoint inhibitors, such as anti-CTLA-4, anti-PD-1 and anti-PD-L1, bypass the immune checkpoint, with the aim of rescuing and enhancing the function of anti-tumor T cells [19].

Immunotherapy or vaccine studies to date have primarily focused on the stimulation of CD8+ T cells and/or T helper (Th)-1 pro-inflammatory responses to biological targets expressed by tumor cells [20]. For example, the high molecular weight tumor-associated glycoprotein, MUC1 (CD227) [21,22,23,24,25], has been used as a target for cancer immunotherapy studies in both mice [26,27,28,29,30,31,32,33,34,35,36,37,38,39,40,41,42,43] and in human clinical trials [44,45,46]. In particular, human clinical studies of MUC1 conjugated to the carrier mannan has been in phase I, II, III trials, and in early stage breast cancer patients these conjugates show protection against recurrence up to 18 years post-vaccination clinical follow-up [47,48,49,50]. Although showing efficacy in early stage breast cancer patients, in other clinical studies in advanced cancer patients clinical outcomes were only modest. In an attempt to improve clinical outcomes, checkpoint inhibitors are being used in combination with vaccines/immunotherapeutics. In addition, it is important to understand the role of other immune cell infiltrates within the TME, which will aid in the design of new improved cancer immunotherapeutics. Herein, we focus on immune cells within the TME and immune-checkpoints associated with breast cancer progression.

## 2. Tumor Microenvironment

**Figure 1:**
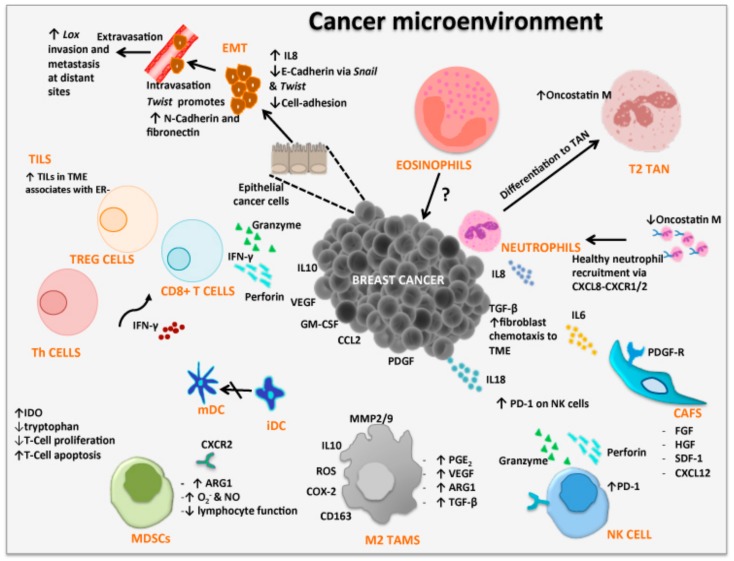
Tumor-associated immune cells in the tumor microenvironment (TME) of breast cancer models. Within the TME there is an array of resident cells contributing to the progression and metastasis of breast cancer cells. The different residents and their associated secretory elements including stimulatory growth factors, chemokines and cytokines are shown. The expression of these residents within the TME of breast cancer patients may aid in discovering new markers associated with specific subtype leading to earlier diagnosis and better clinical outcome. [ARG1. Arginase 1; CAF. Cancer-associated Fibroblast; CD163. Macrophage scavenger receptor; CCL2; Chemokine Ligand 2; CXCL8-CXCR1/2. Chemokine Ligand 8-Chemokine Receptor 1 & 2; CXCL12. Chemokine Ligand 12; COX-2. cyclooxygenase-2; EMT. Epithelial-Mesenchymal Transition; ER− Estrogen Receptor Negative; FGF. Fibroblast Growth Factor; GM-CSF. Granulocyte-Macrophage Colony-Stimulating Factor; HGF. Hepatocyte Growth Factor; IDO. Idoleamine-2, 3-Dioxygenase; IL. Interleukin; iDC. immature Dendritic Cells; mDC. mature Dendritic Cells; MDSCs. Myeloid-derived suppressor cells; M2 TAMS. M2 subtype Tumor-associated Macrophage; NK CELL. Natural Killer; N2 TAN. N2 Subtype Tumor-associated Neutrophil; PDGF-R. Platelet-Derived Growth Factor Receptor; PD-1. Programmed cell death protein 1; PGE_2_. Prostaglandin E2; ROS. Reactive Oxygen Species; SDF-1. Stromal cell-derived factor-1; TGF-β. Transforming Growth Factor-beta; Th Cells. T-helper cells; TILs. Tumor-Infiltrating Lymphocytes; TREG. T-regulatory cells; VEGF. Vascular endothelial growth factor].

Advances in the understanding of the TME in pre-invasive and invasive breast cancer have demonstrated strong evidence to suggest that the TME and associated molecules as well as the infiltration of immune cells, soluble factors and altered extracellular matrix, are involved in promoting tumor growth and metastasis [51,52]. These critical elements of the TME have initiated the identification of new breast cancer markers, such as biological, immunological and immunosuppressive which are associated with tumor development and progression [53]. More recently, there are implications that the nervous system also modulates immune responses in suppressing tumor growth, or, enhances tumors ability to evade immune surveillance leading to metastasis [12,13,14,54]. Over the past decades of cancer research, focus has predominately fallen into the complex interactions of the TME. Its contribution to cancer progression relies heavily on the residents within the TME including, tumor-associated macrophages (TAMs), cancer-associated fibroblasts (CAF), endothelial cells, pericytes, leukocytes, tumor-infiltrating lymphocytes (TILs), cytokine milieu and the extracellular matrix (ECM) (Figure 1, Table 1). It is important to note that tumor cells can efficiently recruit stromal cells (e.g., fibroblasts), immune cells and vascular cells by secreting growth factors, cytokines and chemokines. These cells build the microenvironment by releasing growth promoting signals and intermediate metabolites as well as remodel tissue structure. The signaling between the cancer cells and the TME stimulates or inhibits proliferation and metastatic capability [55,56,57,58]. The tumor not only manages to escape from the host immune system, but it effectively benefits from infiltrating cells by modifying their functions to create the microenvironment favorable to tumor progression [59].

### 2.1. Epithelial-Mesenchymal Transition

Epithelial-mesenchymal transition (EMT) is the ability of cancer cells to leave their epithelial state and acquire characteristics of mesenchymal cells. EMT plays a fundamental role in tumor progression and metastasis and involves a number of signaling pathways such as transforming growth factor beta (TGFβ), nuclear factor-κB (NF-κB), Wnt, Notch and others [60]. EMT markers such as *Snail*, *Twist-1* and *Lox* are transcription factors that induce EMT which have also been shown to be expressed by human breast tumors [61,62]. *Snail* is known to repress *E*-cadherin (a key molecule involved in epithelial adhesion) expression, a hallmark of EMT, and has been linked to the migratory and invasive phenotype of breast cancer cells [63]. *Twist* is a helix loop helix protein involved in the downregulation of the epithelial genes such as E-cadherin, claudins, occludins and stimulates a number of the mesenchymal genes including, N-cadherin and fibronectin [64]. *Lox* is involved in remodeling the extracellular matrix facilitating invasion and metastasis [65]. In addition, circulatory tumor cells and highly enriched for characteristics of mesenchymal cells showing loss of E-cadherin and gain of N-cadherin [66,67]. Studies show that CXC chemokine ligand 8 (CXCL8 or IL-8) promotes the EMT of human breast cancer cells via the formation of the *TWIST1-p65* complex that activates transcription of NF-κB and increases the binding affinity of p65 to CXCL8 [68]. Studies of EMT markers in triple negative breast cancer (TNBC) and non-TNBC subtypes showed the overexpression of *Lox* to be significantly higher in TNBC subtype compared to non-TNBC subtypes. However, the expressions of *Snail* and *Twist* showed no difference between TNBC and non-TNBC [62]. Although the expression of *Lox* cannot be linked to prognosis it may be a possible target for future TNBC systemic therapies (Figure 1, Table 1). In addition, immune infiltrates in TNBC is associated with good prognosis although this is not the case for ER+ tumors.

### 2.2. Tumor-Infiltrating Lymphocytes

Immune cells present within the tumor include those mediating adaptive immune responses, such as T cells, dendritic cells and B cells, as well as effectors of the innate immune responses, i.e., macrophages, neutrophils, eosinophils and natural killer cells [69]. In fact, premalignant ductal breast carcinoma in situ shows an increase in lymphocyte infiltration, with predominant cells being, activated T cells, B cells, and, the immune suppressive regulatory T cells (Treg; CD4+CD25+Forkhead box protein 3 (Foxp3)+ cells) [70]. Tumor-infiltrating lymphocytes (TILs) are largely CD8+ T cells, CD4+ T helper (Th) cells and Treg cells [69,71]. Among CD4+ T cells present in the tumor, a subset of CD4+CD25^high^Foxp3+ Treg cells are able to suppress proliferation of other T cells within the microenvironment through contact-dependent mechanisms, or anti-inflammatory cytokine (IL-10 and TGFβ) secretion [72,73]. Cytotoxic CD8+ T-cells have the ability to kill cancer cells via secretion of pro-inflammatory interferon-γ (IFNγ) and granzyme-perforin complex. In addition, the activation and maturation of CD8+ T-cells is also modulated by IFNγ secreted by CD4+ Th1 cells and specific tumor-associated antigens processed by dendritic cells [74]. In relation to breast cancer subtypes, ER− cancers show higher number of TILs compared to ER+ cancers [75]. In addition, the overall number of T cells, B cells, macrophages and myeloid-derived suppressor cells (MDSC) are also higher in ER− compared to ER+ breast cancers [75]. Thus, the number of TILs within the tumor microenvironment may not only help distinguish subtype but can be used to identify markers associated with progression and metastasis from the primary tumor (Figure 1, Table 1).

### 2.3. Cancer-Associated Fibroblasts

Fibroblast cells are important contributors to the development of the extracellular matrix and they secrete a number of factors including collagen and cytokines, which aids in the structural framework of the extracellular matrix [76]. Within the TME and surrounding space, accumulation of different immune and regulatory cells may stimulate or inhibit tumor growth (Figure 1, Table 1). Fibroblasts represent the majority of stromal cells within the TME. Commonly, activated fibroblasts inhibit early stages of tumor progression through production of fibroblast factors and IL-6 occurring in gap junctions [77]. However, epithelial cells, endothelial cells, and cancer cells have been associated with altering fibroblasts into cancer-associated fibroblasts (CAFs) [78]. CAFs have shown to secrete various growth factors and cytokines associated with promoting breast cancer proliferation and metastasis [79]. In fact, fibroblast growth factor, human growth factor, tenascin, thrombospondin-1, TGFβ and stromal cell-derived factor 1 (SDF-1 or CXCL12), are found in cancer cells including breast cancer, at sites of chronic inflammation produced by CAFs [80,81,82]. The secretion of high levels of TGFβ by tumor cells causes migration of fibroblasts to the TME initiating trans-differentiation of fibroblasts to CAFs [83]. Platelet-derived growth factor can also indirectly recruit myofibroblasts by stimulating TGFβ secretion from macrophages [84]. Thus, CAFs have the potential to promote growth and angiogenesis, remodel the ECM, and direct cell-to-cell interaction. Furthermore, CAFs have been associated with breast cancer subtype, ER+, TNBC and HER2+ [85]. Gene expression analysis have indicated that CAFs within the tumor stroma may have subtype-specific gene expression profiles, with CAFs increasing the invasive properties of the HER2+ subtype. These studies show the importance of CAFs in cancer progression and their ability to alter the normal function of fibroblasts and their anti-tumorigenic abilities. In addition, further studies are warranted to bridge the gap in knowledge as CAFs may hold potential prognostic value and could be used to distinguish breast cancer subtype and stage.

### 2.4. Tumor-Associated Macrophages

Macrophage cells are large phagocytic cells found at sites of inflammation and engulf foreign antigens and cancer-associated proteins to stimulate the adaptive immune responses. Macrophages (and dendritic cells) have been the target for cancer immunotherapy and vaccines studies [86,87,88,89,90]. Macrophages also infiltrate the breast cancer microenvironment to initiate an inflammatory response but differentiate into tumor-associated macrophages (TAMs) resembling M2 macrophages (Figure 1). TAMs are re-programmed to inhibit lymphocyte functions through the secretion of inhibitory cytokines including IL-10, prostaglandins and reactive oxygen species (ROS) [91,92]. ROS activates TGFβ which inhibits cell growth by arresting cells in the G1 phase of the cell cycle leading to either terminal differentiation or induction of apoptosis [93]. TAMs also produce extracellular matrix-degrading enzymes such as metalloproteinases (MMPs). The proteolytic cleavage of TGFβ from latency-associated peptide regulates the secretion, expression and activation of MMP2, MMP3, MMP9 and MMP13, which facilitate tumor cell migration, degradation of blood vessel basal membranes and, metastasis [94,95]. M2 macrophages express arginase 1 (ARG1), anti-inflammatory cytokines, and proteases that support their pro-oncogenic functions [19]. Increased numbers of CD163+ M2 macrophages associate with unfavorable prognosis linked to non-luminal and basal-like breast cancer subtype [96,97]. In addition, vascular endothelial growth factor (VEGF) secretion by TAM stimulate tumor angiogenesis, promoting its invasiveness and metastatic potential [98,99,100]. TAMs are also able to transform cancer cells to undergo EMT which subsequently enhances the invasion and metastasis of breast cancer cells [101,102,103]. Similarly, mesenchymal-like breast cancer cells can activate macrophages to a TAM-like phenotype by granulocyte macrophage-colony stimulating factor 1 (GM-CSF1) [101].

TAMs are also associated with the regulation of programmed death receptor-1 (PD-1) and its ligand (L; PD-L1) expression in the tumor microenvironment of TNBC. IFNγ, IL-1β, tumor TNFα, TGFβ, IL-6, and IL-18 are key to the functionality of TAM [104,105]. The expression of PD-L1 on tumor cells might be induced by the secretion of IFNγ via Janus kinase/signal transducer and activator of transcription 3 (JAK/STAT3) and the phosphatidylinositol 3-kinase (PI3K)/protein kinase B (Akt) signaling pathways, which has been shown in lung cancer [106]. TGFβ increases the suppressive ability of TAMs by differentiating TAMs into M2 macrophages and upregulation of PD-L1 leads to tumor escape [107]. Tumor-derived IL-18 increases the immunosuppressive properties on NK subsets inducing their PD-1 expression which is correlated with poor prognosis of TNBC patients [108]. This mechanism can lead to inactivation of TILs in the TME and cancer immunotherapy and vaccines studies, and would, therefore, not be effective in such an environment.

### 2.5. Tumor-Associated Neutrophils

Neutrophils are polymorphonuclear cells whose main function in the innate immune response is the first line of defense and protection against fungal and bacterial infections. Neutrophils are abundantly found in human blood and migrate to a number of tissues from the blood circulation. In recent years it has been noted that neutrophils are also present within the TME with both pro- and anti-tumorigenic properties [109]. In the TME, cytokines initiate polarization of tumor-associated neutrophils (TANs) causing them to differentiate into either pro-inflammatory/anti-tumorigenic (N1 phenotype) or, anti-inflammatory/pro-tumorigenic (N2 phenotype) (Figure 1, Table 1). Their migration from the blood circulation into the TME is stimulated by IL-8 (CXCL8-CXCR1/2 axis) expression by tumor cells [109]. Neutrophils in their normal state do not secrete oncostatin M however, upon interaction with cancer cells oncostatin M becomes highly expressed in TANs. Oncostatin M exhibits an inhibitory effect on cell proliferation of breast cancer cell lines exerting a pro-inflammatory response by inducing adhesion and chemotaxis of neutrophils [110,111]. Conversely, oncostatin M has also been shown to promote tumor progression by enhancing angiogenesis and metastasis to breast cancer cell lines (MDA-MB-23 and T47D) [112]. Neutrophils isolated from healthy human volunteers display no expression of oncostatin M but upon co-culture with MDA-MB-231 or T47D cells, oncostatin M expression is significantly increased [112]. Furthermore, production of GM-CSF1 by breast cancer cells and cell-cell contact are also necessary for neutrophils to release oncostatin M which has also been noted to concurrently increase VEGF and invasive capacity of cell lines [112].

TANs associate with aggressive breast cancer phenotype, facilitates angiogenesis, promotes mutagenesis and suppresses the immune system, leading to poor prognosis of patients [113,114]. The presence of TAN in breast cancer has also been associated with the clinical subtypes of breast cancer indicating that there is a preferential chemotaxis of neutrophils dependent on the subtype [115]. In fact, TANs predominate in TNBC compared to non-TNBC, suggesting that factors associated with the TME may have direct or indirect effects on neutrophil production and chemotaxis into the TME [115]. In addition, TGFβ which is highly expressed in TNBC has been shown to be a major contributor to neutrophil chemotaxis, however, TGFβ may also induce a pro-tumorigenic N2 phenotype [116,117,118]. The presence of TANs in the TME of breast cancer patients highlights their involvement in tumor growth and metastasis. However, not much is known regarding their presence and their correlation to the range of breast cancer subtypes, and whether they impact in cancer immunotherapy studies.

### 2.6. Tumor-Associated Eosinophils

The primary function of eosinophils was believed to be to fight parasitic and bacterial infections, and were involved in the pathogenesis of inflammatory diseases such as allergic asthma and chronic obstructive pulmonary disease with high secretion of IL-5 and other eosinophilia granules [119]. More recently, evidence suggests that eosinophils are abundant in inflammatory bowel disease although their role within the intestinal tissues is not clear [120]. Blood eosinophils migrate to tissues at sites of infection. In the human breast, eosinophils are crucial for mammary gland development where they are interlaced within the terminal end buds of the breast together with a complex stroma of fibroblasts and macrophages; once terminal end buds convert into terminal end ducts, fibroblasts disappear [121,122]. Eosinophils have also be observed within the TME of cancer, including colon, ovarian, prostate and lung carcinomas [123] (Figure 1). Of interest, eosinophils have been noted at the edge of breast cancer biopsy wounds, suggesting that breast cancer biopsies may trigger the recruitment of inflammatory cells including eosinophils [124]. In addition, low eosinophil counts in the peripheral blood of breast cancer patients is a major risk factor of breast cancer recurrence [123]. The presence and amount of mast cells and tumor-associated eosinophils was determined in patients diagnosed with invasive breast cancer of different stages [125]. In that study, it was noted that eosinophils were also not present within the TME. Although a positive correlation of improved prognosis with tumor-associated eosinophils has been shown in a number of solid cancers, in Hodgkin’s lymphoma their presence in the TME display a poor prognosis [123]. There is conflicting data on the presence of eosinophils within the TME of breast cancer which requires further studies to understand their role and interaction with other immune cells in the TME and their interaction with cancer cells. The little or lack of eosinophil presence in breast cancers compared to other solid tumors, could be associated with the current methods utilized or different interactions between eosinophils and breast cancer that are yet not defined. Whether eosinophils contribute to immune suppression or immune stimulation in breast cancer immunotherapy studies is also unknown.

### 2.7. Myeloid-Derived Suppressor Cells

Myeloid-derived suppressor cells (MDSCs) are a diverse population of myeloid progenitor cells which have been shown to play a key role in chronic inflammation and cancer development [126]. In cancer, MDSCs (CD34+CD33+CD13+CD15(−)) promote tumor cell growth and suppresses immune cell function through production of arginase 1 (ARG1) which synergizes with inducible nitric oxide synthase (iNOS) to increase superoxide and nitric oxide (NO) production, thus, reducing lymphocyte function [127,128] (Figure 1, Table 1). Studies on the levels and phenotypes of myeloid cells in peripheral blood and TME of breast cancer patients revealed high levels of tumor-infiltrating myeloid cells, including granulocytes and immature cells lacking expression markers for fully differentiated monocytes or granulocytes [129]. However, the expansion was not reflected in the peripheral blood or in samples from non-breast cancer patients. In addition, the presence of ARG1 expression, important in T cell suppression, reinforces the immunosuppressive ability of MDSCs [129]. Furthermore, MDSCs isolated from breast cancer tissues show high expression of indole amine 2,3 dioxygenase (IDO), an enzyme responsible for the catabolism of tryptophan. This depletion of tryptophan by IDO in the TME produces kynurenine based by-products that lead to inhibition of T cell proliferation and induces T cell apoptosis [130].

## 3. Immune Checkpoint Molecules and Breast Cancer

**Figure 2:**
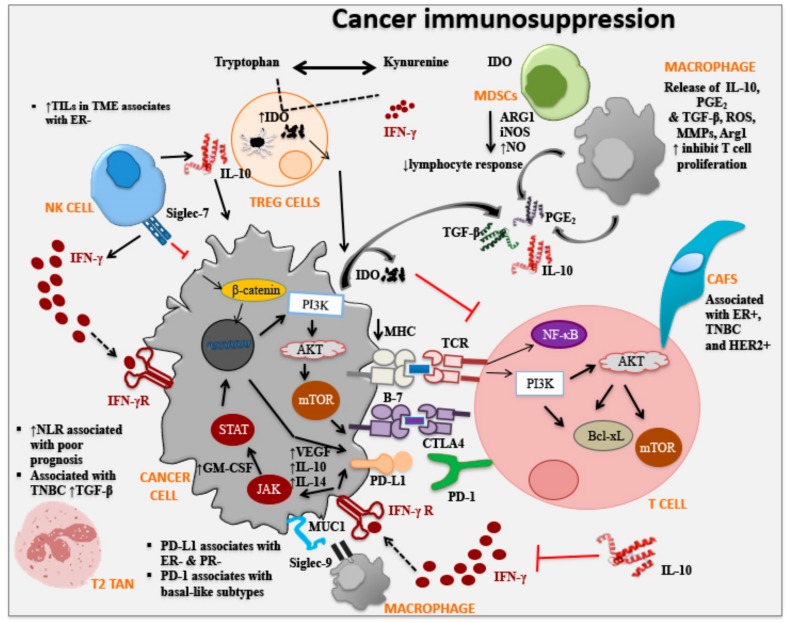
Immune checkpoints of immunosuppressive actions associated with breast cancer. Immune checkpoints of inhibitory pathways are fundamental in the immune system to maintain self-tolerance and modulate immune responses. In breast cancer, some of these immune checkpoints and immunosuppressive factors have been associated with subtype specificity through their expressions on breast cancer cells. The different immune cells and their ligand-receptor interactions and secreted stimulatory growth factors, chemokines and cytokines are shown. The expression of these immune markers in the TME in breast cancer may or not be subtype-specific but are important in circumventing immune recognition or to immobilize effector T cells. Thus, the expression of these ligands and receptors may be associated with breast cancer stage and clinical outcome. (AKT. serine/threonine kinase or protein kinase B; ARG1. Arginase 1; Bcl-xL. B-cell lymphoma-xtra large; CAFs. Cancer-associated fibroblasts; CTLA4. cytotoxic T-lymphocyte-associated protein 4; ER− Estrogen Receptor Negative; GM-CSF. Granulocyte-Macrophage Colony-Stimulating Factor; HER2+. Human Epidermal Growth Factor Receptor 2; IDO. Indoleamine-2,3-dioxygenase; IFN-γ. interferon gamma; IFN-γR. interferon gamma receptor; IL. Interleukin; JAK. Janus kinase; iNOS. Inducible nitric oxide synthase; MDSC. Myeloid-derived suppressor cells; MHC. major histocompatibility complex; MMP. Matrix Metalloproteinases; mTOR. Mammalian target of rapamycin; MUC. Mucins; NK. natural killer; NF-κB. nuclear factor-κB; NLR. Neutrophil-lymphocyte ratio; NO. Nitric Oxide; PI3K. PI3K.phosphoinositide 3-kinase; PD-1. programmed death-1; PD-L1. Programmed death-ligand1; PGE_2_. Prostaglandin E2; PR−. Progesterone Receptor Negative; ROS. Reactive Oxygen Species; SHP. Src homology protein-tyrosine phosphatase; Siglec 9. Sialic acid-binding lectins 9; STAT. Signal transducer and activator of transcription; TAMs. Tumor-associated macrophages; TANs. Tumor-associated neutrophils; TGF-β. Transforming Growth Factor-beta; TIL. Tumor-Infiltrating Lymphocytes; TNBC. Triple Negative Breast Cancer; Treg. regulatory T cell; TCR. T cell receptor; VEGF. Vascular endothelial growth factor).

Immune-editing involves the process of malignant cell progression based on cancer cell and immune cell interactions in three stages: (i) Elimination: where cancer cells are eliminated following immuno-surveillance involving both innate and adaptive immune cell infiltration including, NK cells, NK-T cells, T cells, and increased pro-inflammatory cytokines in the TME; (ii) Equilibrium: the balance between anti-tumor and tumor-promoting factors in that transformed cells are held in control but are not eliminated following immuno-surveillance; and (iii) Escape: where modifications to tumor cells themselves shape disease progression, as seen in breast cancer, where escape mechanisms include reduced expression of major histocompatibility complex class I [39] and/or co-stimulatory molecules and increased expression of immunosuppressive factors [16]. Immunosuppressive factors involved in the evasion of immune-surveillance play influential roles in cancer progression. Immune checkpoints of inhibitory pathways are crucial for the immune system to maintain self-tolerance and modulate immune responses in order to minimize damage. Immune checkpoints (such as CTLA4, PD-1/PD-L1, IDO, and Siglec-9) are initiated by ligand-receptor interactions and can be blocked by antibodies or modulated by recombinant forms of ligands or receptors [131] (Figure 2, Table 2). It is important to understand immune checkpoint molecules and their role in breast cancer and numerous clinical trials are assessing the efficacy of immune checkpoint inhibitors (via monoclonal antibodies) in cancer therapies either alone or in combination with other cancer therapies.

### 3.1. Cytotoxic T Lymphocyte-Associated Protein 4

Cytotoxic T lymphocyte-associated protein 4 (CTLA-4, CD152), expressed on the surface of activated T cells and a subset of Treg cells, functions as an immune checkpoint molecule which can downregulate T cells and inhibit anti-tumor responses [132]. CTLA-4 interacts with the cell surface immune stimulatory markers CD80 and CD86 on dendritic cells, resulting in the activation of dendritic cells and CD4/CD8 T cells. Treg cells (CD4+Foxp3+) are able to block this interaction resulting in decreased dendritic cell activation, inhibition of cytokine production (IL-2), T cell cycle arrest and suppression of CD8+ T cell proliferation [132]. One approach cancer cells have evolved to escape from the immune system, is via the expression of CTLA-4 on their surface and in a limited number of studies, it has been shown to decrease survival in non-small lung cancer and nasopharyngeal cancer patients [133]. In vitro, co-culture of monocyte-derived human dendritic cells activated with lipopolysaccharide and CTLA-4+ breast cancer cell line, resulted in decreased expression of MHC class II, and costimulatory cell surface molecules (CD40, CD80, CD83, CD86) on dendritic cells and inability to activate T cells [133]. The addition of anti-CTLA-4 monoclonal antibody was able to reverse dendritic cell suppression and activate CD8+ T cells and induce apoptosis of CTLA-4+ breast cancer cells. Thus, CTLA-4 blockade is a promising approach for therapeutic studies in breast cancer patients, with or without combination with immunotherapy. Further, CTLA-4 expression by T cells is associated with better prognosis, whereas the expression of CTLA-4 on tumor cells is associated with poor prognosis [134].

### 3.2. Programmed Cell Death Protein 1

Programmed cell death protein 1 (PD-1, CD279) which is homologous to CD28, is an inhibitory immune signaling molecule and regulates adaptive immune responses. PD-1 is not expressed on circulating T cells but is expressed on activated T cells via TGFβ, IL-2, IL-7, IL-15 and IL-21. PD-1 is also expressed by activated NK cells, B cells, monocytes, dendritic cells, myeloid cells, and thymocytes. The ligands (L) for PD-1 are PD-L1 (B7-H1, CD274) and PD-L2 (B7-DC, CD273) which are expressed on dendritic cells and monocytes and are upregulated by IFNγ, GM-CSF, vascular endothelial growth factor, VEGF, lipopolysaccharide, IL-10 and IL-14 [133]. The interaction between PD-1 and its ligands PD-L1 or PD-L2 leads to decreased T cell activity and its expression has been associated with poor prognosis [135]. In breast cancer, in particular in TNBC subtype, PD-L1 is upregulated which suppresses tumor-infiltrating T cells [136] resulting in higher Ki-67+ cells, basal-like subtypes, and distant metastasis [137]. Furthermore, analysis of PD-L1 expression in breast cancer cell lines showed higher PD-L1 expression in basal and mesenchymal cell lines than luminal cell lines [138]. Clinical samples from breast cancer patients, show upregulated expression of PD-L1 and is associated with poor prognostic features: ductal type, large tumor size, high grade, ER−, PR−, ERB-B2 receptor tyrosine kinase 2 (ERBB2)+, high proliferation rate and aggressive molecular subtypes (basal and ERBB2-enriched) [138]. In the last decade a number of clinical trials have been conducted to block PD-L1/PD-1 axis on non-small cell lung carcinoma and melanoma [135]. In fact, as of March 2019 the FDA approved an anti-PD-L1 antibody, in combination with chemotherapy, for the treatment of triple-negative, metastatic breast cancer for patients whose tumors expressed PD-L1. 

### 3.3. Indoleamine 2,3-Dioxygenase

Indoleamine 2,3-dioxygenase (IDO) is an enzyme which catalyzes the oxidative break-down of tryptophan via kynurenine pathway in the presence of IFNγ [139]. Tryptophan is a vital amino acid for cell survival, and a lack of tryptophan in the TME leads to inhibition of T cell proliferation, thus, IDO exerts an immunosuppressive effect allowing tumor cells to escape from immune cells [130,139,140]. There are a number of mechanisms by which IDO influences the anergy of T cell activity within the TME. Firstly, by inhibiting mammalian target of rapamycin complex 1 and protein kinase C, both of which are regulators of glucokinase (GLK1), an important glucose regulator [141,142]. Secondly, the expression of IDO on cancer cells can activate the general control non-depressible-2 (GCN2) inducing a stress response in cells. GCN2 is stimulated by tryptophan transfer RNA (tRNA) in cells from accumulated uncharged tryptophan as a result of tryptophan degradation via IDO. Stimulation of GCN2 by the tryptophan tRNA alters protein translation and, prevents activation of T cells and promotes Treg cell differentiation [143,144,145]. Thirdly, the production of kynurenine, resulting from catabolism of tryptophan, activates transcription of the aryl hydrocarbon receptor (AHR), leading to differentiation of Foxp3+ Treg cells which suppress of anti-tumor responses [146,147]. Expression of high levels of IDO by cancer cells has been correlated with poor prognosis and reduced overall survival in patients with solid tumors including, breast cancer [140]. In fact, microvesicles released by malignant tumors, in breast cancer patients contained a significantly high expression of IDO compared to normal breast tissues or benign tumor samples [148,149]. In addition, IDO expression is higher in advanced stages of breast cancer and mainly expressed in the TNBC subgroup [148,149]. The expression of IDO in TNBC has also shown to correlate with high expressions of PD-L1 in invasive primary breast carcinomas which highlights the need for combination therapies over single ones [150].

### 3.4. Sialic Acid-Binding Immunoglobulin-Type Lectin

Sialic acid-binding immunoglobulin-type lectins (Siglecs) are expressed on the surface of immune cells and to date, 15 different Siglecs have been identified. In particular Siglec-7 on NK cells and Siglec-9 on neutrophils, dendritic cells, monocytes, are associated with anti-tumor immunity [151]. In particular, Siglec-9 interacts with sialic acids on cancer cells (i.e., with MUC1, a high molecular weight glycoprotein expressed on adenocarcinoma cells including breast cancer) leading to immune suppression [152]. The mucous barrier that protects the epithelia contains secreted and transmembrane mucins. These membrane-bound mucins have important biological roles in cell-cell and cell-matrix interactions [20,25]. Human adenocarcinomas overexpress tumor-associated transmembrane mucins, such as MUC1 and MUC4, and are associated with tumor progression by enhancing their role in cell growth and survival [153,154]. Thus, mucins have been identified as potential prognostic and therapeutic targets for breast cancer development. In fact, vaccines/immunotherapeutic strategies targeting MUC1 have shown promise in pre-clinical animal models and in human clinical trials, with immune cell activation and clinical responses [24,25,42,48,155,156,157,158,159,160,161]. MUC1 is cleaved into N- and C- terminal subunits (MUC1-N and MUC1-C) which form a heterodimeric complex expressed at the cell membrane [162]. Studies have shown that MUC1-C functions as an oncoprotein by interacting with epidermal growth factor receptor (EGFR), ERbB2, and other tyrosine kinase receptors [163]. MUC1 also contributes to the malignant phenotype of cancer cells by binding to β-catenin, blocking phosphorylation and degradation [163,164]. In fact, the interaction between Siglec-9 and MUC1 via sialylated *O*-glycans, aids in cell growth by stimulating the recruitment of β-catenin [152]. In breast cancer, overexpression of MUC1 is associated with poor prognosis [165], suggesting that Siglec-9 may play an important role in the progression of breast cancer. Hence, studies that evaluate the complex interaction between Siglec-9-MUC1 and breast cancer, as well as the mechanism of this interaction in vitro and in vivo are warranted. In addition, human clinical studies utilizing MUC1 as a target should include blockade of Siglec-9 for improved clinical outcomes to patients with breast and other cancers. In fact, at the American Associate for Cancer Research Meeting in Chicago April 2018, it was announced that first-in-class antibodies against Siglec-9 immune checkpoints are being developed for cancer immunotherapy (Benac, O., et al., conference presentation).

## 4. Conclusions and Future Prospects

The interaction between the immune system and the TME have become a pivotal point of research. The ability of cancer cells to signal pro-tumorigenic modifications to normal anti-tumorigenic cells to aid in cell proliferation and angiogenesis has provided multiple advancements in cancer therapies and prognostic value. As a consequence, not all current therapies and treatments have been able to provide relief from the 100 or more types of cancers, with breast cancer being the most common among women. In addition, in breast cancer, each subtype and stage has risk factors for incidence, treatment response, rate of disease progression and metastasis associated with it [53]. Current studies continue to isolate the different immune cell populations and those found within the TME of breast cancers, their association with hormone receptor phenotype and immune-evasion mechanisms. The interaction between immune cells and cancer cells in the TME and immune-checkpoints could potentially be used for identification of new markers associated with progression of tumors in their early developmental stages.

Currently, breast cancer-positive hormone receptor phenotypes (ER+, PR+, HER2+) have more treatment options with favorable outcomes compared to the TNBC subtype. The immune cells function and their cytokines are key factors whose modulation strongly encourages further study and consideration as predictive markers and important therapeutic targets in different subtypes of breast cancer [183]. No systemic method has been associated with the combination of markers in early diagnosis of cancer patients. Although the current methods and biomarkers can be used to place patients within a clinical stage critical for treatment options, having a systemic method that correlates the expression of biological, immunological, and potential neurological markers could pave the way into new prognostic and clinical outcomes of cancer patients. In addition, all this combined with immune checkpoint inhibitors and cancer immunotherapeutics would yield new improved treatments for breast cancer.

## Figures and Tables

**Table 1:** Cells within the tumor microenvironment and their role in breast cancer.

Cell Type	Mechanisms	Model	Detection	Ref
Epithelial-mesenchymal transition (EMT) (via transcription factors *Snail*, *Twist-1* and *Lox* and markers vimentin and N-cadherin)	Involved in tumor progression and metastasis through signaling pathways such as TGFβ, NF-κB, Wnt, Notch			[60,61]
	*Lox* important in extracellular matrix (ECM) → invasion and metastasis	Triple negative breast cancer (TNBC) and non-TNBC human samples	Immunohistochemistry (IHC)	[62]
	↑*Lox* in TNBC			
	NF-κB → ↑*Snail* → TNF-α induced EMT	MCF-7 Cell line	Cell migration (CM)	[166]
			Real time polymerase chain reaction (RTPCR)	
			Western Blot (WB)	
	MSC’s (Mesenchymal stem cells) ↑metastasis through facilitation of EMT	MDA-MB-231, T47D and SK-Br_3_ cell lines	Low-density array	[167]
			RT-PCR	
			Gene expression and proliferation assays	
Immune cells Tumor-infiltrating Lymphocytes (TILs), T-cells (Tregs: CD4, CD25, FOXP3; CD8+; CD4+ Th cells)	Suppress T-cell proliferation			[74]
	Induce tumor cell death via IFN-γ and granzyme-perforin molecules			
	naïve CD4+ T cell recruitment → ↓Immunosuppression	MDA-MB-231 cell line	IHC and immunofluorescence staining, Flow cytometry (FC), Migration assay	[70]
		Primary breast carcinoma		
CD4+ naïve T cell		Female NOD/scid mice	qRT-PCR, Binding assays, WB	
		Humanized mice NOD/SCID/IL2rγ^null^ (NSG)		
Tumor-associated	TAMs are re-programmed to inhibit lymphocyte functions through release of inhibitory cytokines such as IL-10, prostaglandins or reactive oxygen species (ROS)			[91,92]
macrophage (TAM)	↑CD163+ in non-luminal and basal-like breast caner	Human tumor tissue	IHC with CD163	[96]
	breast cancer cell-secreted factors modulate macrophage differentiation to M2 status	Human tumor tissue	IHC	[168]
			FC	
		Cell line MCF-7, MDA-MB231 and T47D	ELISA	
			Zymography	
	↑CD163+ in tumor stroma of TNBC	Human Luminal A and Triple Neg/basal-like tissue	IHC	[169]
			Gene Expression	
Cancer-associated Fibroblast (CAF)	Shown to secrete various growth factors and cytokines associated with promoting breast cancer proliferation			[79]
	CAFs derived from Her2+ breast cancers → ↑actin cytoskeleton and integrin signaling	Breast tumors sub grouped according to receptor expression	IHC	[85]
			Gene Expression	
	ER+ expressing CD146^neg^ → ↑tumor resistance to tamoxifen	Human tissue (Stage II & III, ER+ and/or ER−)	Immunocytochemistry (ICC)	[170]
	ER+ expressing CD146^pos^ → ↓tumor resistance to tamoxifen	MCF-7 cell line	Gene expression	
	TNBC exhibit CAF subsets, ↑CAF-S1 → ↑T Lymphocyte survival → ↑Treg → Ø T effector proliferation → Immunosuppression	Female BC patient cohort (Luminal, HER2 and TN subtype tissues)	FC	[171]
			IHC	
Tumor-associated Neutrophils (TANs)	N2 phenotype: pro-tumorigenic or pro-inflammatory			[172]
	N1 phenotype: anti-tumorigenic			
	Oncostastin M expressed by TANs → ↑angiogenesis and metastasis	MDA-MB-231 & T47D cell lines	ICC	[112]
			ELISA	
	↑TAN in TNBC	Stage I-III breast cancer patient tumors divided into three subtypes: hormone-receptor [HR]-positive, HER2-negative (HR+, HER2-ve); HER2-positive and triple negative (TN)	Hematoxylin & eosin	[115]
			IHC	
	↑TβRIII (TGF-β receptor) in TNBC → ↑mesenchymal-stem like (MSL) TNBC cells → cell migration, invasion, and tumorigenicity	MSL cell lines SUM159, MDA-MB-231 and MDA-MB-157	Cell proliferation assay	[116]
			CM and invasion assay	
			Immunoblotting	
			FC	
			Gene Expression	
Tumor-associated Eosinophils	High presence of eosinophils at biopsy site may be linked to proliferation rate of tumor cells adjacent to wound	Female patients with primary breast cancer	Peripheral eosinophil counts	[173]
Myeloid-derived suppressor cells (MDSCs)	↑Arginase 1 (ARG1) + nitric oxide synthase (iNOS) → ↑superoxide and nitric oxide (NO) → Ø lymphocyte responses → ↑iNOS in surrounding cells → ↑tumor growth and ↓ immune cell functions			[127,128]
	stage IV patients with extensive metastasis → ↑MDSC	Blood from patients with stages I–IV solid malignancies obtained prior to surgery	FC	[174]
	↑MDSC correlates with worse prognosis	Peripheral blood specimens stage IV breast cancer patients	FC	[175]
			Proliferation assay	
	MDSC ↑IDO → ↓tryptophan → Ø T-cell proliferation and induced T-cell apoptosis	Female breast cancer patients (Stages I–III)	IHC	[176]
			RT-PCR	
			WB	
			ELISA	

**Table 2:** Immune Checkpoints and Breast Cancer.

Immune Checkpoint Factor	Mechanisms	Model	Detection	Ref
**CTLA4**	Expressed on the surface of activated T-cells and a subset of Tregs ↓T-cell activation → Anti-T cell response			[177]
	CTLA4 blockade → Ø proliferation and induced apoptosis of CTLA-4+ breast cancer cells	MDA-MB-231, SKBR3, MCF-7 and T47D cell lines	FC	[178]
			WB	
	↑CTLA-4 in lymphocytes → better prognosis	130 BC patients	IHC	[134]
	↑CTLA-4 in T cells → worse prognosis			
**PD-1 (PD-L1/PD-L2)**	PD-1 is expressed by activated lymphocytes → ligation PD-L1/PD-L2 → ↓T-cell activity → poor prognosis			[135]
	TNBC subtype; ↑PD-L1 → suppresses auto-immunity—T cell proliferation—Cytokine production—Cytotoxic activity			[136]
	PD1 ↑TILs, but ↓PDL1 in T cells → positive TNBC prognostic factor	negative ER, PR, and HER-2 BC patients	IHC	[137]
			RNAscope	
	↑PD-L1 in Basal & TNBC subtypes → ↑cytotoxic local immune response → ↑better survival	BC patient is Cell lines		[138]
**Indoleamine 2,3-dioxygenase (IDO)**	Catalyzes the oxidative break-down of tryptophan via kynurenine pathway in the presence of IFN-γ → enabling immune escape			[130]
	Correlation between expression of IDO and PD-1 in myoepithelial, stromal, and T cells	Human BC patient tissues and healthy tissues	IHC	[179]
			WB	
			RT-PCR	
	↑IDO expression in TNBC and basal-like BC	200 TNBC patients	IHC	[180]
			RT-PCR	
	IDO expression ↑ER+ as compared to ER− is	breast cancer tissue sections	IHC	[52]
**Siglec-9**	Siglec-9 found on neutrophils and Siglec-7 found on NK cells have been associated with anti-T immunity			[151]
	Siglec-9 → T-associated MUC1 downstream signal transduction, following T cell proliferation	Transgenic mice and murine mammary T cell	WB, Immunoperoxidase staining, IHC	[181]
			Gene expression	
			Proliferation (MTT) assay	
	↑Siglec-9 on DCs involved in immunoregulation through ligation with mucins in epithelial cancer	Human colon cancer cell line (LS 180 cells)	FC	[182]
			RT-PCR	
			ELISA	
